# Targeting MCR-3 and membrane biosynthesis: mechanistic insights into pterostilbene-colistin synergy

**DOI:** 10.1128/spectrum.02588-25

**Published:** 2026-03-17

**Authors:** Fei Zeng, Wenjuan Yin, Huilian Duan, Shuxin He, Luying Sun, Jie Zhang, Siming Wang, Feng Liu, Yuangong Zhang

**Affiliations:** 1 Key Laboratory of Pathogenesis Mechanism and Control of Inflammatory Autoimmune Disease of Hebei Province, School of Basic Medicine Science, Hebei University 56667, Baoding, China; Universita degli Studi dell'Insubria, Varese, Italy

**Keywords:** pterostilbene, colistin, MCR-3, membrane disruption, antibiotic adjuvant

## Abstract

Colistin is a crucial antibiotic for treating infections caused by multidrug-resistant Enterobacterales. However, the emergence of plasmid-mediated *mcr* genes has significantly compromised their clinical efficacy. Natural adjuvants, such as pterostilbene, offer a promising strategy to restore antibiotic efficacy against resistant bacteria. This study aims to elucidate the molecular mechanisms underlying the synergistic action between pterostilbene and colistin (polymyxin E) against *mcr-3*-positive *Escherichia coli*. The *in vitro* synergistic activity between pterostilbene and colistin was confirmed by checkerboard assays. This effect was further supported by bacterial viability and membrane potential analysis. In the *Galleria mellonella* infection model, combination therapy also demonstrated therapeutic efficacy. Molecular docking revealed that pterostilbene directly targets key catalytic residues (H380 and E111) of the MCR-3 enzyme, inhibiting its phosphoethanolamine transferase activity, a finding validated by site-directed mutagenesis. Transcriptomic analysis further revealed that pterostilbene downregulated genes involved in fatty acid biosynthesis and central carbon metabolism while upregulating fatty acid degradation pathways. These metabolic disruptions were associated with compromised membrane integrity, as confirmed by electron microscopy and membrane permeability assays. Supplementation with exogenous phospholipids attenuated the synergistic effect, supporting a lipid-mediated mechanism. This study demonstrates that pterostilbene enhances colistin efficacy through dual mechanisms: direct inhibition of MCR-3 enzymatic function and disruption of bacterial membrane stability. These findings provide a strong theoretical foundation for developing pterostilbene-based adjuvants to combat *mcr*-mediated colistin resistance.

**IMPORTANCE:**

Colistin remains one of the last therapeutic options for infections caused by multidrug-resistant Enterobacteriaceae, but plasmid-borne *mcr* genes threaten their utility. We identified that the natural compound pterostilbene restores polymyxin activity against *mcr-3*-positive *Escherichia coli* through dual mechanisms: direct inhibition of MCR-3 catalytic residues and disruption of membrane stability via metabolic reprogramming. These dual actions compromise resistance and enhance bacterial killing both *in vitro* and *in vivo*. Our findings provide mechanistic insight into a plant-derived molecule that could be developed to counteract colistin resistance, highlighting a promising approach for extending the lifespan of critical last-line antibiotics.

## INTRODUCTION

The widespread dissemination of multidrug-resistant (MDR) bacteria has made infection control increasingly challenging (1, 2). In 2021, over one million deaths were attributed to antimicrobial resistance (AMR), a number projected to rise to 8.22 million by 2050, with an estimated 1.91 million deaths directly caused by bacterial AMR (3). Colistin, once abandoned due to nephrotoxicity and neurotoxicity (4), was reintroduced into clinical practice in 2017 as a “last-resort treatment” against MDR gram-negative bacteria infections (5). However, the emergence of plasmid-borne *mcr* genes has undermined their effectiveness (6). Consequently, identifying novel strategies to overcome *mcr*-mediated resistance has become a pressing global challenge. MCRs confer resistance by modifying lipid A through phosphoethanolamine (pEtN) transferase activity, reducing colistin binding. To date, 10 *mcr* variants have been identified, all sharing similar resistance mechanisms (7). Given the extensive diversity and high-risk potential of *mcr-3* variants, this study focuses on *mcr-3* as a representative resistance gene.

The development of novel antibiotics is time-consuming and costly, and the continual evolution of bacterial resistance further complicates this process (8). As an alternative, antibiotic adjuvants have emerged as a promising strategy to prolong the clinical utility of existing antibiotics. These compounds, though lacking direct antibacterial activity at low concentrations, can potentiate antibiotic efficacy or counteract bacterial resistance mechanisms (9). Their advantages include restoring antibiotic susceptibility in MDR strains, delaying resistance development, and reducing both antibiotic dosage and associated toxicity (10, 11). Compared to new antibiotic development, adjuvant discovery is considered more cost-effective. These adjuvants are derived from diverse sources, including plant- and animal-derived natural products (12–14), synthetic substances (15–18), and other potential adjuvants (19, 20). Among them, plant-derived natural products are particularly attractive due to their structural diversity, multitarget actions, low toxicity, and synergistic potential with antibiotics (21–23).

Stilbenes, a class of natural compounds broadly distributed in dietary plants, possess broad biological activities, including antioxidant, anti-inflammatory and anticancer properties (24, 25). As antibiotic adjuvants, stilbene compounds offer advantages, such as low toxicity and the ability to enhance antibiotic efficacy and reverse bacterial resistance (26, 27). Stilbene monomers exhibit diverse chemical modifications, resulting in substantial chemical complexity and enhanced biological activity (28). Dimeric stilbenes show notable synergistic effects with antibiotics, particularly those targeting bacterial protein synthesis. This synergy boosts antimicrobial potency of antibiotics and may help prevent bacterial resistance development (29).

Recent studies have demonstrated that pterostilbene, a naturally stilbene derivative, exhibits strong synergistic effects with colistin against *mcr-*positive *Escherichia coli* (30). This highlights its potential as an effective antibiotic adjuvant. In this study, pterostilbene was selected as a model stilbene compound to investigate its antibacterial mechanisms. By examining both changes in bacterial resistance phenotypes and underlying resistance mechanisms, we aim to elucidate how pterostilbene enhances antimicrobial activity. These findings offer new insights into the development of novel stilbene-based antibiotic adjuvants for combating MDR bacterial infections.

## RESULTS

**Fig 1:**
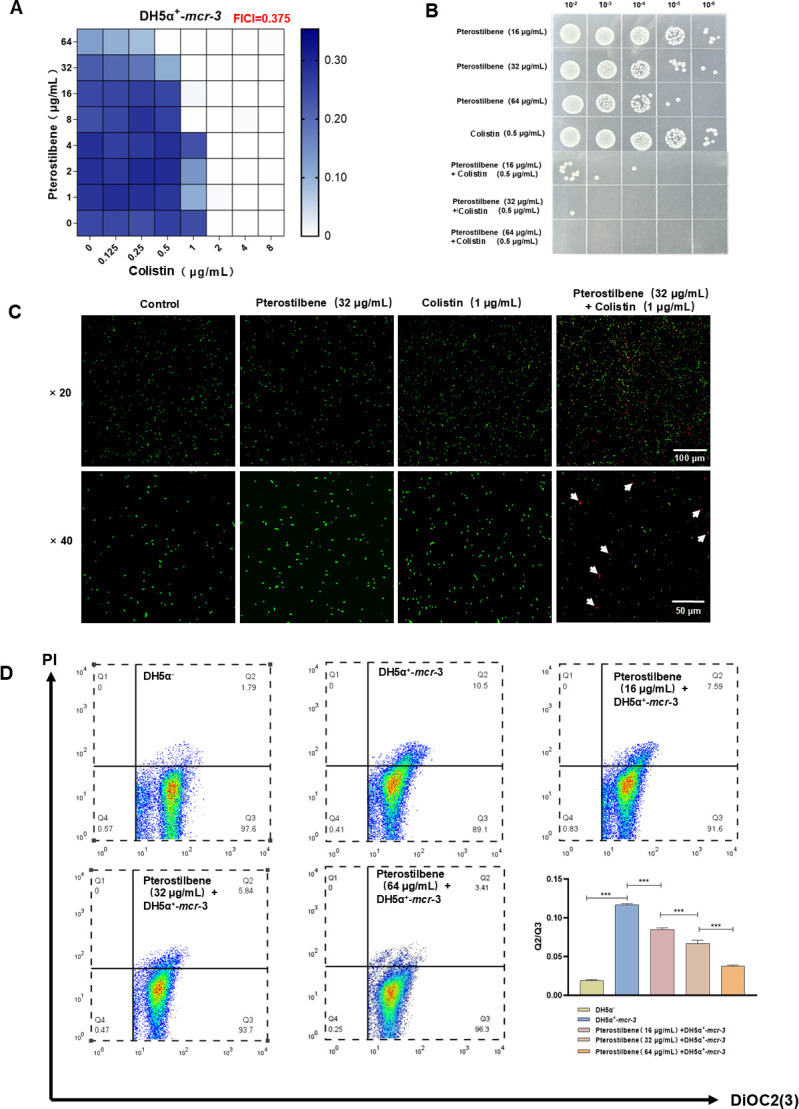
(**A**) Synergistic effect of pterostilbene combined with colistin against DH5α**^+^**-*mcr-3*. (**B**) Bactericidal activity determined by droplet plate counting. (**C**) Confocal laser scanning microscopy images of DH5α**^+^**-*mcr-3* under monotherapy and combination treatments. (**D**) The membrane potential-sensitive fluorescent dye DiOC_2_(3) assessing pterostilbene-induced changes in membrane potential. Quantitative flow cytometry analysis showing changes in the membrane potential of DH5α**^+^**-*mcr-3* after pterostilbene treatment (*n* = 3; ***P < 0.001).

To evaluate the synergistic antibacterial activity of pterostilbene and colistin, the checkerboard microdilution method was used to assess their combined effect against colistin-resistant *E. coli* DH5α**^+^**-*mcr-3*. The results showed that at a pterostilbene concentration of 64 μg/mL, the combination exhibited significant synergy, with a fractional inhibitory concentration index (FICI) value of 0.375 (Fig. 1A), consistent with previous findings (30). We further examined the synergistic effect of pterostilbene with colistin against *mcr*-positive strains carrying other variants. The results revealed that pterostilbene exerted comparable synergistic effects with colistin against these strains; the corresponding data are provided in Fig. S1. Meanwhile, pterostilbene was also able to restore colistin susceptibility in *mcr-3*-harboring *Aeromonas salmonicida*, as shown in Fig. S2.

Further confirmation was provided by droplet plate counting and live/dead staining assays. When pterostilbene was combined with a sub-inhibitory concentration of colistin (0.5 μg/mL), the antibacterial activity was markedly enhanced in a dose-dependent manner (Fig. 1B and C). In the *Galleria mellonella* larval infection model with DH5α**^+^**-*mcr-3*, combination therapy increased the survival rate to 92.9% compared with monotherapy (Fig. S3). Giemsa-stained hemolymph smears revealed that combination treatment significantly reduced the aggregation of phagocytes in the hemolymph post-infection, indicating a lower bacterial burden (Fig. S4). These results demonstrate that the pterostilbene-colistin combination is effective in eliminating DH5α**^+^**-*mcr-3*. Mechanistically, MCR-3 reduces the net negative charge of the bacterial membrane by catalyzing the addition of pEtN to lipid A, thereby diminishing colistin binding and resistance. Membrane potential analysis using the DiOC_2_(3) probe revealed that pterostilbene treatment significantly restored the membrane potential of DH5α**^+^**-*mcr-3* cells in a concentration-dependent manner (Fig. 1D). This suggests that pterostilbene can reverse the MCR-3-mediated membrane potential disruption, acting as an effective antibiotic adjuvant to restore colistin susceptibility in resistant bacteria.

**Fig 2:**
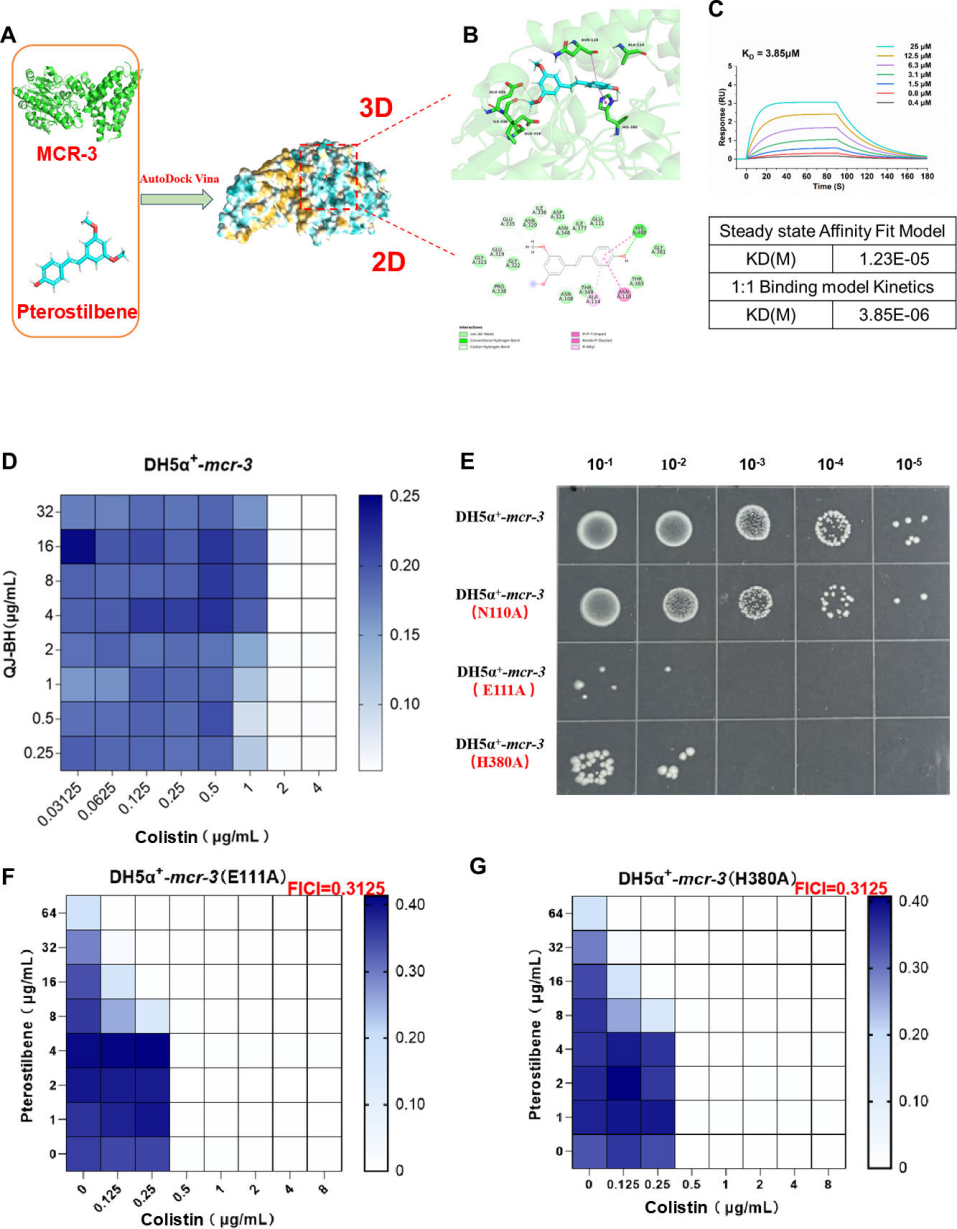
(**A**) Schematic diagram of the molecular docking results between pterostilbene and the MCR-3 protein. (**B**) 3D and 2D interaction diagrams illustrating binding interactions between pterostilbene and MCR-3. (**C**) Surface plasmon resonance sensorgrams obtained from Bcthi4WT-coated chips at different concentrations of pterostilbene (**D**) *In vitro* bactericidal activity of the QJ-BH and colistin combination against DH5α**^+^**-*mcr-3*. (**E**) Bactericidal effects of monotherapy and combination therapy on mutant strains assessed by droplet plate counting. (**F**) Synergistic effect of pterostilbene and colistin against the DH5α**^+^**-*mcr-3* (E111A) mutant strain. (**G**) Synergistic effect of pterostilbene and colistin against the DH5α**^+^**-*mcr-3* (H380A) mutant strain.

To elucidate the molecular mechanism by which pterostilbene reverses antibiotic resistance, molecular docking between pterostilbene and the MCR-3 protein was performed using AutoDock Vina 1.2.5 (Fig. 2A). The binding energy was −8.012 kcal/mol, indicating strong binding affinity (31). The 2D and 3D interaction diagrams revealed that pterostilbene interacts with MCR-3 primarily through hydrogen bonding, hydrophobic interactions, and van der Waals forces (Fig. 2B). Furthermore, we successfully expressed and purified the MCR-3 protein and confirmed its direct interaction with pterostilbene using surface plasmon resonance (SPR) analysis. The steady-state affinity fit model yielded a dissociation constant (Kd) of 12.3 μM, whereas the 1:1 binding kinetics model gave a Kd of 3.85 μM, both indicating a specific and moderate binding affinity between MCR-3 and pterostilbene (Fig. 2C). The hydroxyl group of pterostilbene formed stable hydrogen bonds with the active site residue HIS380 (H380) of the MCR-3 protein. To verify the role of the hydroxyl group, QJ-BH was synthesized by protecting the hydroxyl group of pterostilbene (Fig. S5). QJ-BH exhibited no synergistic antibacterial effect when combined with colistin (Fig. 2D), suggesting that the hydroxyl moiety is critical for the antibiotic adjuvant activity of pterostilbene. This phenotypic observation corroborates the docking results.

Moreover, the aromatic ring of pterostilbene interacted with the active site residue GLU111 (E111) through van der Waals forces and with HIS380 via hydrophobic interactions (Fig. 2B), potentially contributing to the inhibition of MCR-3 activity. To further confirm this, site-directed mutagenesis of E111 and H380 residues resulted in complete loss of MCR-3 function (Fig. 2E). Matrix-assisted laser desorption/ionization-time of flight mass spectrometry (MALDI-TOF-MS) analysis of lipid A from the mutant strains revealed the absence of the characteristic 1920 *m*/*z* peak associated with MCR-3-mediated pEtN modification. This confirmed that E111 and H380 are essential catalytic residues of MCR-3 (Fig. S6). Interestingly, the mutant strains exhibited an even stronger synergistic response to the combination of pterostilbene and colistin (Fig. 2F and G), suggesting that pterostilbene may act not only by targeting MCR-3 directly but also through additional mechanisms or alternative cellular targets.

**Fig 3:**
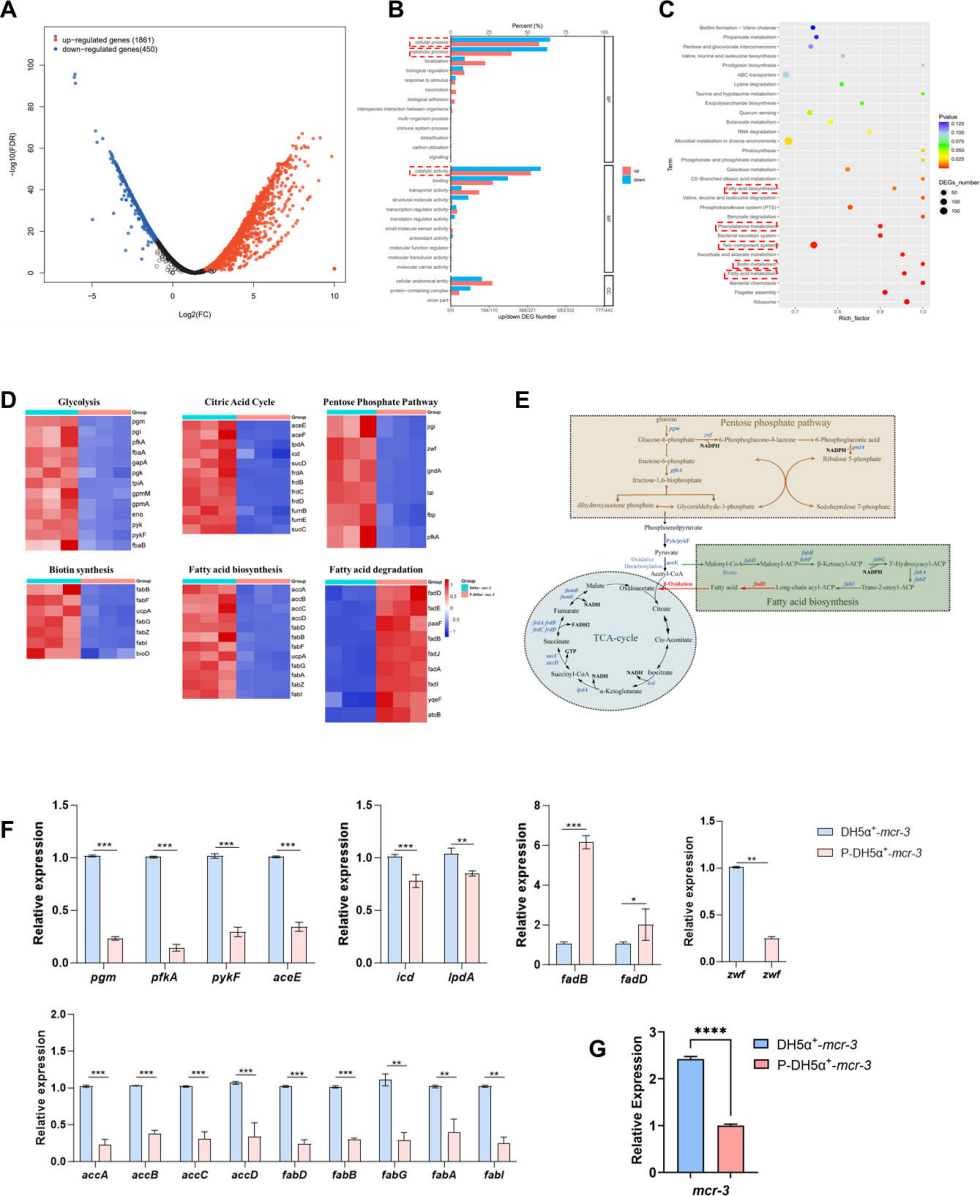
(**A**) DEGs following pterostilbene treatment. (**B**) GO enrichment analysis of DEGs, categorized into BP, CC, and MF. (**C**) KEGG pathway enrichment analysis of DEGs. (**D**) Heatmap showing expression patterns of selected DEGs. (**E**) Fatty acid biosynthesis pathway. Genes marked in red are upregulated, while those in blue are downregulated after treatment. (**F**) Transcriptional levels of key genes involved in the fatty acid biosynthesis pathway in DH5α**^+^**-*mcr-3* before and after pterostilbene treatment. (**G**) Transcriptional level of *mcr-3* genes in DH5α^+^-*mcr-3* before and after pterostilbene treatment. (*n* = 3; *P < 0.05, **P < 0.01, ***P < 0.001, ****P < 0.0001).

To explore additional potential mechanisms underlying the adjuvant activity of pterostilbene, a prokaryotic transcriptomic analysis was conducted to assess its impact on gene regulation in *E. coli*. The volcano plot results revealed that pterostilbene significantly altered the gene expression profile of MCR-3-positive *E. coli* (Fig. 3A). Gene Ontology (GO) annotation indicated that pterostilbene affected cellular processes, metabolic pathways, and catalytic activities (Fig. 3B). KEGG enrichment analysis further revealed significant changes in pathways related to fatty acid biosynthesis and metabolism, biotin metabolism, two-component regulatory systems, and ribosome function, among others (Fig. 3C and D).

Based on these findings, we identified that pterostilbene enhances the bactericidal effect of colistin against DH5α^+^-*mcr-3* by disrupting bacterial metabolic functions. Specifically, key genes involved in fatty acid biosynthesis (*accA/B/C/D* and *fabA/B/I/G*) were significantly downregulated following pterostilbene treatment (Fig. 3E). In addition, key genes in glycolysis and the tricarboxylic acid cycle (*pgm*, *pfkA*, *pykF*, *aceE*, *zwf*, *icd*, and *lpdA*), which provide the NADPH and energy required for fatty acid synthesis, were also markedly downregulated (Fig. 3F). Suppression of these pathways likely impairs fatty acid synthesis. Conversely, genes involved in fatty acid degradation, such as *fadB* and *fadD*, were significantly upregulated, indicating a shift toward fatty acid breakdown. This combined effect, reduced synthesis and enhanced degradation of fatty acids, ultimately disrupts phospholipid biosynthesis and compromises membrane integrity. Importantly, pterostilbene also inhibited the transcription of the *mcr-3* gene, thereby attenuating *mcr-3*-mediated colistin resistance at its source (Fig. 3G).

**Fig 4:**
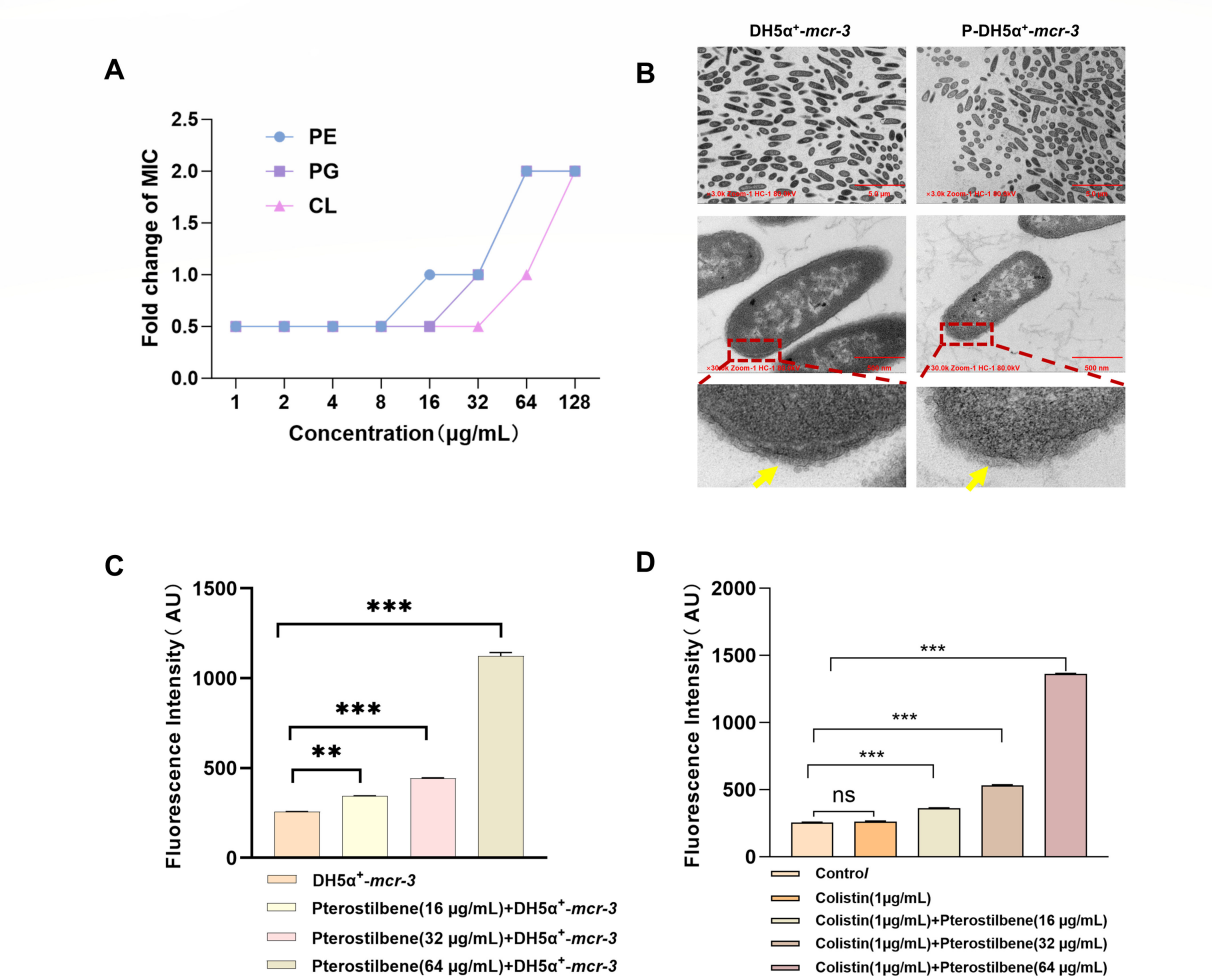
(**A**) Exogenous addition of phosphatidylglycerol (PG), phosphatidylethanolamine (PE), and cardiolipin (CL) abolished the adjuvant activity of pterostilbene. (**B**) Transmission electron microscopy images showing morphological alterations in DH5α**^+^**-*mcr-3* after pterostilbene treatment. (**C**) Changes in outer membrane permeability of DH5α**^+^**-*mcr-3* upon treatment with pterostilbene alone. (**D**) Changes in outer membrane permeability of DH5α**^+^**-*mcr-3* upon co-treatment with pterostilbene and colistin (*n* = 5; **P < 0.01, ***P < 0.001; ns, not significant).

Furthermore, the addition of exogenous phospholipids significantly reduced the bactericidal activity of colistin against DH5α^+^-*mcr-3* in a dose-dependent manner (Fig. 4A), supporting the hypothesis that interference with fatty acid and phospholipid metabolism weakens membrane stability, thereby restoring bacterial susceptibility to colistin. Transmission electron microscopy revealed that treatment with 32 μg/mL pterostilbene resulted in the bilayer membrane structure appearing less clearly defined (Fig. 4B). Consistent with this, N-phenyl-1-naphthylamine (NPN) fluorescence assays demonstrated that pterostilbene increased outer membrane permeability in a concentration-dependent manner (Fig. 4C). Moreover, when combined with colistin, pterostilbene further exacerbated membrane damage in MCR-3-positive *E. coli* (Fig. 4D). Collectively, these results demonstrate that pterostilbene disrupts fatty acid biosynthesis and compromises membrane integrity, thereby significantly restoring colistin sensitivity in DH5α**^+^**-*mcr-3*.

## DISCUSSION

Antibiotic resistance is rapidly emerging as a global public health crisis. In particular, the widespread dissemination of multidrug resistance among gram-negative bacteria has led to a significant decline in the efficacy of many conventional antibiotics. Against this backdrop, the exploration of non-antibiotic compounds as antibiotic adjuvants has become a key direction in combating resistance.

These adjuvants can precisely disrupt resistance mechanisms, enhance the efficacy of existing antibiotics, and extend their clinical utility (9). Natural compounds are known not only for their intrinsic antibacterial activity but also for their ability to enhance antibiotic efficacy through various mechanisms. Numerous studies have demonstrated that stilbene compounds can enhance host defense mechanisms by modulating immune responses and reprogramming bacterial metabolic pathways, thereby exhibiting greater potential for *in vivo* antimicrobial therapy (32, 33). In this study, we focused on the active natural compound pterostilbene, derived from traditional Chinese medicine, and systematically evaluated its potential as an antibiotic adjuvant in reversing MCR-3-mediated colistin resistance. We further investigated its effects on bacterial membrane lipid metabolism and associated resistance pathways.

Previous studies have shown that pterostilbene exhibits strong synergistic antibacterial activity with colistin against *E. coli* strains carrying the *mcr-3* gene (30). However, the underlying mechanism behind this synergy has remained unclear. MCR-3 catalyzes the addition of pEtN groups to lipid A in the outer membrane of gram-negative bacteria, reducing the net negative charge of lipid A and weakening its affinity for colistin, thereby conferring resistance (7). In this study, we found that pterostilbene significantly enhanced colistin’s antibacterial efficacy (FICI = 0.375), restoring outer membrane potential and increasing colistin sensitivity by fourfold, thereby reversing resistance (Fig. 1). Although several known antibiotic adjuvants have been reported to modulate membrane surface charge (34), our findings provide further evidence that pterostilbene acts primarily through direct interaction with the MCR-3 protein to restore membrane potential (Fig. 2; Fig. S4). Based on this, we propose that other natural compounds targeting MCR-family proteins may also hold potential as adjuvants to colistin.

Natural compounds also restore colistin sensitivity in gram-negative bacteria by depleting lipid biosynthesis and altering phospholipid composition (35, 36). Our findings suggest that pterostilbene’s ability to sensitize resistant bacteria to colistin not only may be solely due to its interaction with MCR-3 but also may involve effects on other molecular targets or biological pathways in *E. coli*. Through prokaryotic transcriptomic analysis, we discovered that pterostilbene downregulates key genes involved in fatty acid biosynthesis, disrupts the supply of precursors, energy, and reducing agents, promotes fatty acid degradation, and interferes with phospholipid synthesis (Fig. 3). These alterations compromise membrane structure and function, increase permeability, and ultimately reverse bacterial resistance (Fig. 4). Therefore, pterostilbene exhibits great potential for antimicrobial applications due to its dual mechanism of action: simultaneously inhibiting MCR-3 protein activity and disrupting membrane lipid homeostasis.

These findings provide a solid scientific foundation for the development of novel antibiotic adjuvants and highlight the significant scientific and therapeutic potential of plant-derived compounds in combating antibiotic resistance.

### Conclusions

This study demonstrates that pterostilbene restores colistin sensitivity in *mcr*-3-positive *E. coli* through dual mechanisms. First, pterostilbene directly targets the active site of the MCR-3 enzyme, inhibiting its phosphoethanolamine transferase activity. Second, it disrupts bacterial lipid metabolism by downregulating genes involved in fatty acid biosynthesis and energy production while upregulating fatty acid degradation pathways. This metabolic reprogramming compromises membrane integrity, enhancing colistin-mediated bactericidal activity. These findings highlight pterostilbene as a promising “natural adjuvant” for overcoming plasmid-mediated colistin resistance.

## MATERIALS AND METHODS

### Revival and cultivation of bacterial strains

The *E. coli* strain used in this study was DH5α derivatives carrying the empty vector pHSG299 (DH5α**^−^**) or the recombinant plasmid pHSG299-*mcr-3* (DH5α**^+^**-*mcr-3*) (37). These two strains share an identical genetic background, differing only in the presence or absence of the *mcr-3* gene. For selective growth, the strains were streaked onto LB agar plates supplemented with kanamycin (50 mg/L) and incubated overnight at 37°C.

### Minimum inhibitory concentration assays and synergy assessment using the checkerboard assay

The minimum inhibitory concentrations of the compounds were determined using the checkerboard assay (13). The interaction between pterostilbene and colistin was evaluated by calculating the FICI, using the formula:


$$FICI=\frac{MIC\left(A\;in\;combination\right)}{MIC\left(A\;alone\right)}+\frac{MIC\left(B\;in\;combination\right)}{MIC\left(B\;alone\right)}.$$

FICI was interpreted as follows: FICI ≤0.5 indicated synergistic effects; 0.5 < FICI ≤ 4 represented additive/indifferent effects; and FICI >4 denoted antagonistic effects (38).

### Colony counting by droplet plating method

*E. coli* was grown to log phase, adjusted to 10⁶ CFU/mL, and then introduced into culture systems with test compounds. After incubation, the cultures were serially diluted in Mueller-Hinton broth (MHB), and 10 μL of each serial dilution was carefully spotted onto plates for CFU counting after incubation at 37°C.

### Bacterial live/dead staining assay

Log-phase DH5α^+^-*mcr-3* cells were divided into four conditions: untreated control group, pterostilbene-treated group, colistin-treated group, and combination treatment group. After cultivation, *E. coli* cells were harvested for viability using the LIVE/DEAD BacLight Bacterial Viability Kit (Cat. No. L7012; Thermo Fisher Scientific, Waltham, MA, USA).

### *G. mellonella* larval infection model and survival assay

*G. mellonella* larvae (250–300 mg) were infected with *E. coli* (10⁶ CFU/mL), and 10 μL of the suspension was injected into the last pair of prolegs of each larva. After infection, larvae were randomly assigned to five treatment groups: untreated control, PBS, colistin (0.5 mg/kg), pterostilbene (128 mg/kg), and combination therapy group, administered with a total volume of 10 μL. Survival was monitored at 24 and 48 h post-infection. At each time point, hemolymph was collected from randomly selected infected larvae. Hemolymph was collected for Giemsa-stained cytological analysis.

### Measurement of bacterial membrane potential

*E. coli* DH5α^+^-*mcr-3* and DH5α^−^ (control) were incubated at 37°C for 4 h. Following incubation, each group was stained with the membrane potential-sensitive fluorescent dye DiOC_₂_(3) at a final concentration of 30 μM. Staining was performed in the dark at room temperature for 30 min (39). Membrane potential changes were detected using a flow cytometer (FACSCalibur; BD Biosciences, San Jose, CA, USA), and data were analyzed using FlowJo software.

### Molecular docking analysis

The 3D structure of MCR-3 was predicted using AlphaFold 3 based on the *mcr-3* gene sequence. The chemical structure of pterostilbene was retrieved from PubChem (https://pubchem.ncbi.nlm.nih.gov/). Both the MCR-3 (receptor) and pterostilbene (ligand) were processed using AutoDock Tools 1.5.7. Molecular docking was performed using AutoDock Vina 1.2.5, targeting key amino acid residues in the MCR-3 active pocket (E238, T277, H375, D450, H451, N103, T107, E111, G322, K325, H380, and H463) (31). Molecular docking using AutoDock Vina with default parameter (grid spacing = 0.375 Å, exhaustiveness = 8). The prepared protein and ligand files were imported into AutoDock Vina, and the docking calculation was executed.

### SPR analysis

SPR experiments were performed using a Biacore 1K system (Cytiva, Marlborough, USA) to evaluate protein-ligand interactions. The sensor chip surface was first activated by injecting a freshly mixed solution of N-hydroxysuccinimide and 1-ethyl-3-(3-dimethylaminopropyl) carbodiimide (100 μL each) at a flow rate of 10 μL/min. The target protein was diluted to 40 μg/mL in 10 mM sodium acetate buffer (pH 4.0) and immobilized onto the activated chip at 10 μL/min, yielding an immobilization level of approximately 7,000 response units. Residual active esters on the chip surface were blocked using ethanolamine-HCl.

Binding experiments were conducted using the Kinetics/Affinity Wizard. Analytes were injected over the immobilized surface at a flow rate of 30 μL/min, with a contact time of 90 s and a dissociation time of 120 s. Measurements were performed at 25℃ using HBS-EP^+^ as the running buffer. Data were analyzed using Biacore Insight Evaluation Software.

### Pterostilbene modification experiment

For synthesis of compound QJ-BH, pterostilbene (300 mg, 1.17 mmol), potassium carbonate (194 mg, 1.4 mmol), and iodomethane (110 μL, 0.47 mmol) were dissolved in acetone (30 mL). The reaction mixture was heated to 50°C under reflux for 5 h. After cooling to room temperature, the mixture was filtered, and the solvent was removed under reduced pressure using a rotary evaporator to obtain the crude product, QJ-BH. The crude product was purified by silica gel column chromatography (eluent: dichloromethane) and was collected to afford the desired compound QJ-BH.

### Site-directed mutagenesis of MCR-3

Based on molecular docking results, key binding residues of pterostilbene within the MCR-3 protein, GLU111 and HIS380 at the catalytic site and ASN110 outside the active site, were individually mutated to alanine to investigate their functional roles (40). Mutant constructs (GLU111A, HIS380A, and ASN110A) were generated in the pHSG299-*mcr-3* plasmid using site-directed mutagenesis. The resulting plasmids were subsequently transformed into *E. coli* competent cells. Primer sequences used for mutagenesis are listed in Table S1.

### Lipid A extraction and analysis

Log-phase cultures of DH5α^+^-*mcr-3*, DH5α^+^-*mcr-3* (E111A), and DH5α^+^-*mcr-3* (H380A) strains were harvested, washed, and resuspended in LB broth, followed by heat inactivation at 80°C for 1 h. Cells were resuspended in 200 μL distilled water and mixed with 200 μL of 2% acetic acid, followed by heating at 100°C for 30 min. After centrifugation (17,000 × *g*, 2 min), the supernatant was discarded. Pellets were then washed with distilled water, centrifuged again, and resuspended in 50 μL of distilled water for subsequent MALDI-TOF-MS analysis (41).

### RNA sequencing and bioinformatics analyses

DH5α**^+^**-*mcr-3* strains were cultured to log phase, centrifuged, and resuspended in MHB. A 20 mL aliquot was treated with 32 μg/mL pterostilbene (P-DH5α^+^-*mcr-3*) and incubated at 37°C, 200 rpm, for 4 h. Cells were harvested by centrifugation (5,000 rpm, 10 min), washed with PBS, flash-frozen in liquid nitrogen, then stored at −80°C. High-quality total RNA was extracted; RNA-seq libraries were prepared and sequenced using the Illumina NovaSeq 6000 platform at Biozeron Co., Ltd. (Shanghai, China).

Raw reads were first filtered, aligned to the reference genome using STAR v2.4.0b, and quantified with HTSeq-count v0.6.1p1. Differentially expressed genes were identified using the TCC package, with DEGES/edgeR normalization. Functional enrichment was performed using Gene Ontology (BP, CC, and MF) and KEGG pathway analysis, with pathways meeting *P* < 0.01 considered significant.

### Exogenous phospholipid supplementation experiment

The phospholipids phosphatidylglycerol, phosphatidylethanolamine, and cardiolipin were individually dissolved in a solvent mixture (V_DMSO_:V_anhydrous ethanol_ = 2:1). These phospholipid solutions were then thoroughly mixed with pterostilbene (membrane disruption-enhanced photodynamic therapy against gram-negative bacteria by a peptide-photosensitizer conjugate). The phospholipid-pterostilbene mixtures were subsequently combined with varying concentrations of colistin and co-incubated at 37°C for 18–24 h.

### Analysis of membrane permeability

DH5α^+^-*mcr-3* in the logarithmic growth phase was collected and adjusted to a concentration of 10⁶ CFU/mL using PBS. The bacterial suspensions were incubated at 37°C for 3 h with pterostilbene at various concentrations, colistin, or their combination. The outer membrane permeability was evaluated using the fluorescent probe NPN at a final concentration of 10 μM (42). NPN fluorescence was measured at an excitation wavelength of 350 nm and an emission wavelength of 420 nm.

### Transmission electron microscopy analysis

Following the transcriptomic treatment protocol, bacterial samples from the experimental group were washed twice with PBS and fixed overnight at 4°C in 2.5% glutaraldehyde. The cells were then post-fixed in 1% osmium tetroxide, followed by a graded ethanol dehydration series. Samples were embedded in Epon resin using an infiltration protocol and polymerized at 60°C to form solid blocks. Ultrathin sections were prepared and subsequently stained with uranyl acetate and lead citrate to enhance contrast.

### RT-qPCR analysis

Following the transcriptomic experimental protocol, total RNA was extracted from treated *E. coli* DH5α^+^-*mcr-3* cultures. RNA concentration and purity were verified using a NanoDrop spectrophotometer. The MonScript RTIII Super Mix with dsDNase (Two-Step) kit (Monad Biotech) was used for trace DNA removal and reverse transcription following the manufacturer’s protocol. The MonAm ChemoHS qPCR Mix kit (Monad Biotech) was used for quantitative real-time PCR analysis on a Roche LightCycler system (Switzerland), with primer sequences listed in Table S2.

### Statistical analysis

All data are presented as mean ± standard error of the mean. Statistical analyses were performed using SPSS Statistics 27 (IBM). One-way analysis of variance followed by Tukey’s post hoc test was used for comparisons between groups. Graphs were generated using GraphPad Prism 10.0. A *P* value of <0.05 was considered statistically significant.

## Data Availability

The RNA sequencing data generated in this study have been deposited in the NCBI Gene Expression Omnibus database under accession number PRJNA1377225.

## SUPPLEMENTAL MATERIAL

Supplemental material spectrum.02588-25-s0001.docx Tables S1 and S2; Fig. S1 to S6.

ASM does not own the copyrights to Supplemental Material that may be linked to, or accessed through, an article. The authors have granted ASM a non-exclusive, world-wide license to publish the Supplemental Material files. Please contact the corresponding author directly for reuse.
