# Supplementary material for: Targeting MCR-3 and membrane biosynthesis: mechanistic insights into pterostilbene-colistin synergy
Source: Microbiol Spectr. 2026 Mar 17;14(4):e02588-25. doi: 10.1128/spectrum.02588-25 (PMC13055306; doi:10.1128/spectrum.02588-25)
Supplement: Supplemental material — Tables S1 and S2; Fig. S1 to S6. [file spectrum.02588-25-s0001.docx]

Targeting MCR-3 and Membrane Biosynthesis: Mechanistic Insights into Pterostilbene-Colistin Synergy

*Fei Zeng, Wenjuan Yin^*^, Huilian Duan, Shuxin He, Luying Sun, Jie Zhang, Siming Wang, Feng Liu, Yuangong Zhang**

Key Laboratory of Pathogenesis Mechanism and Control of Inflammatory Autoimmune Disease of Hebei Province, School of Basic Medicine Science, Hebei University, Baoding 071002, China

*Author to whom correspondence should be addressed; E-Mail: yinathebei@163.com; [zhangyuangong@hbu.edu.cn](mailto:zhangyuangong@hbu.edu.cn).

**Table S1** **Primer sequences used for site-directed mutagenesis**

| Primer name | Sequence (5’-3’) |
| --- | --- |
| N110A-F | AATCAAGCGGAGGCGTTAGCATATTTAAGCTTACC |
| N110A-R | GCTAACGCCTCCGCTTGATTGGTTTCAAAAATATTCTG |
| E111A-F | CAAAATGCAGCGTTAGCATATTTAAGCTTACCAA |
| E111A-R | GCTAACGCTGCATTTTGATTGGTTTCAAAAATATTCTG |
| H380-F | ACCTGATAGGTAGCGCGGGCCCAACCTACTACAAGC |
| H380-R | GGCCCGCGCTACCTATCAGGTGGAAGCCAACCAGCT |

**Table S2 Primers used for qRT-PCR**

| Primer name | Sequence (5’-3’) |  |
| --- | --- | --- |
| pgm-F | GATGACTGACAACGGCTGGTTC |  |
| pgm-R | CAATCTGCTTGCGATGTTCTTCAC |  |
| pfkA-F | GGCACTGACTACACTATCGGTTTC |  |
| pfkA-R | TACGCTGGTGAGAAGAAGAGGTG |  |
| pykF-F | GGTTGCGAACAAGGCGTAGAC |  |
| pykF-R | GCTTTCAGGTGCTCACGGATTTC |  |
| aceE-F | | GGCACAGGTATCAGCAACTACATC |
| aceE-R | | CGGCGTTCCAGTTCCAGATTAC |
| icd-F | | CGGCACAAGGCAAGAAGATCAC |
| icd-R | | ACACCGATTCCATCACCTTCAATG |
| lpdA-F | | CGGTGGTCTGGCTGGTATGG |
| lpdA-R | | GCCGTTCTCACCTTCAACTTCC |
| zwf-F | | CTTGGTAAAGAAACGGTGCTGAAC |
| zwf-R | | CCACTTCTTCTGCCACGGTAATC |
| accA-F | AAGCCATTGCACGCAACCTG |  |
| accA-R | ACCGCCAGAACCACCTTCAC |  |
| accB-F | GCCAGCAGCAGCGGAAATC |  |
| accB-R | CGTTGACTTTCTGACCCACTTCG |  |
| accC-F | CGGGTTACGGCTTCCTCTCC |  |
| accC-R | TCAGGCGAATGGTTTCTGCTTTC |  |
| accD-F | CCTTTATGGGCGGTTCAATGGG |  |
| accD-R | CCACCAGAGGCGGAGAAGC |  |
| fabD-F | CTGGTGTGATTGATTTCGCTGATG |  |
| fabD-R | GACCGATGATTGCCGCCATAG |  |
| fabB-F | CGTGAAGTGTTCGGCGATAAGAG |  |
| fabB-R | CGTGTTCCAGCATCAGCAGAG |  |
| fabG-F | GACCAGCGTGCGGGTATCC |  |
| fabG-R | TCGTCGGATGCCAGGAATGC |  |

**Table S2 Primers used for qRT-PCR (continued)**

| Primer name | Sequence (5’-3’) | |  |  |
| --- | --- | --- | --- | --- |
| fabA-F | | | GGCGAAGTGCTGGTTGATGG | |
| fabA-R | | | AGAAGGCAGACGTATCCTGGAAC | |
| fabI-F | CGATTCGCCGTACCGTTACTATTG | |  |  |
| fabI-R | AATGCTGAAACCGCCGTCAAC | |  |  |
| fadB-F | AACTGGCTGGCGTGATCTCC | |  |  |
| fadB-R | ACAACCGCTTCTACCACAATATCC | |  |  |
| fadE-F | CACCGATTGCCATCACCGTTG | |  |  |
| fadE-R | TCGCCGCTTCCATCTCTTCC | |  |  |
| 16S-F | CGAAGACGCCGAAAGCATTT | |  |  |
| 16S-R | CACGATCGTCAACGCCTTTC | |  |  |

**Note:** 16S rRNA was used as the internal reference gene to calculate gene expression changes in this study.


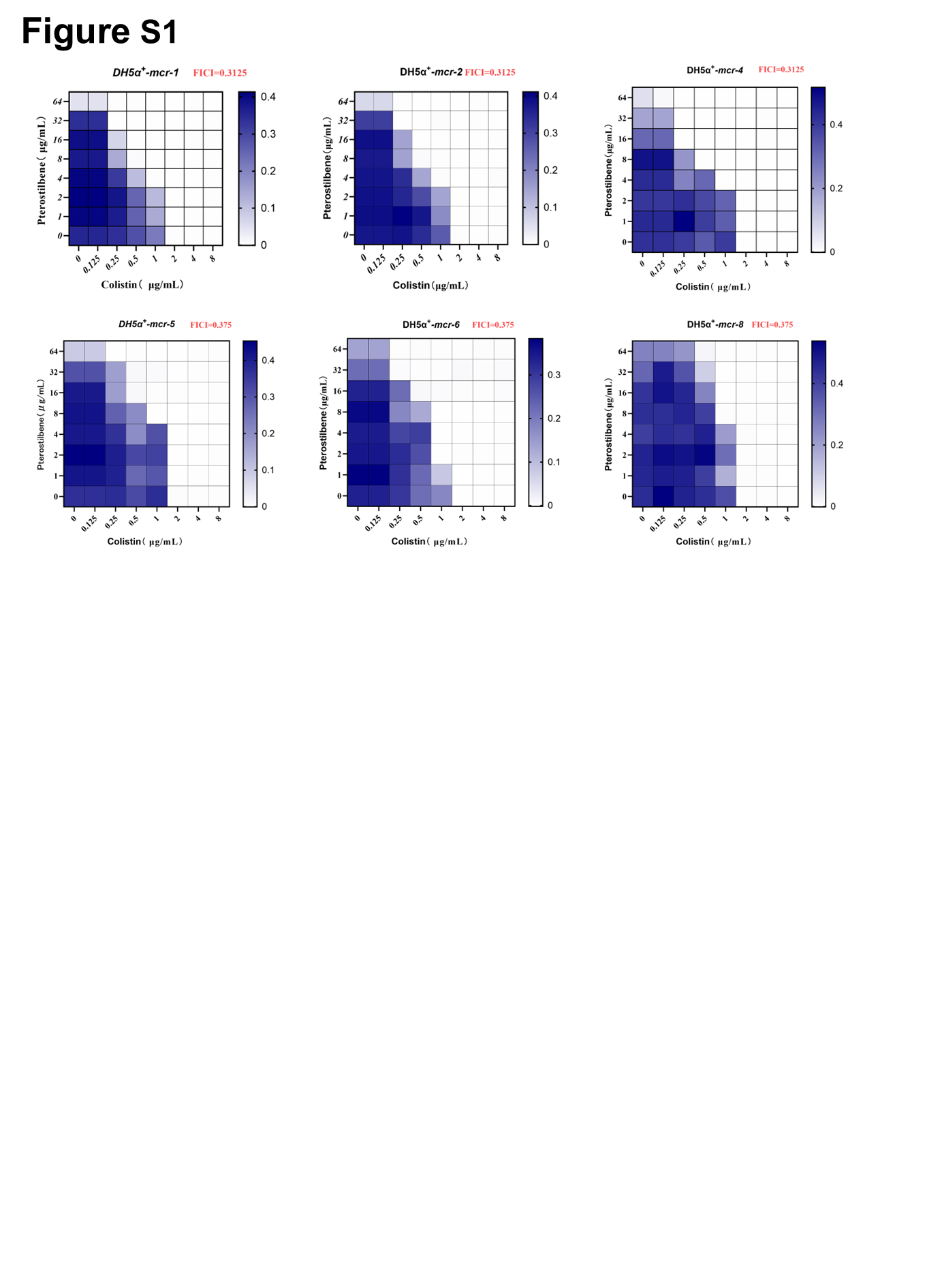


Figure S1. Synergistic effect of pterostilbene combined with colistin against *E. coli* harboring *mcr* variants

Figure S2. Synergistic effect of pterostilbene combined with colistin against *Aeromonas*

*salmonicida* strain AS1 harboring *mcr-3* gene


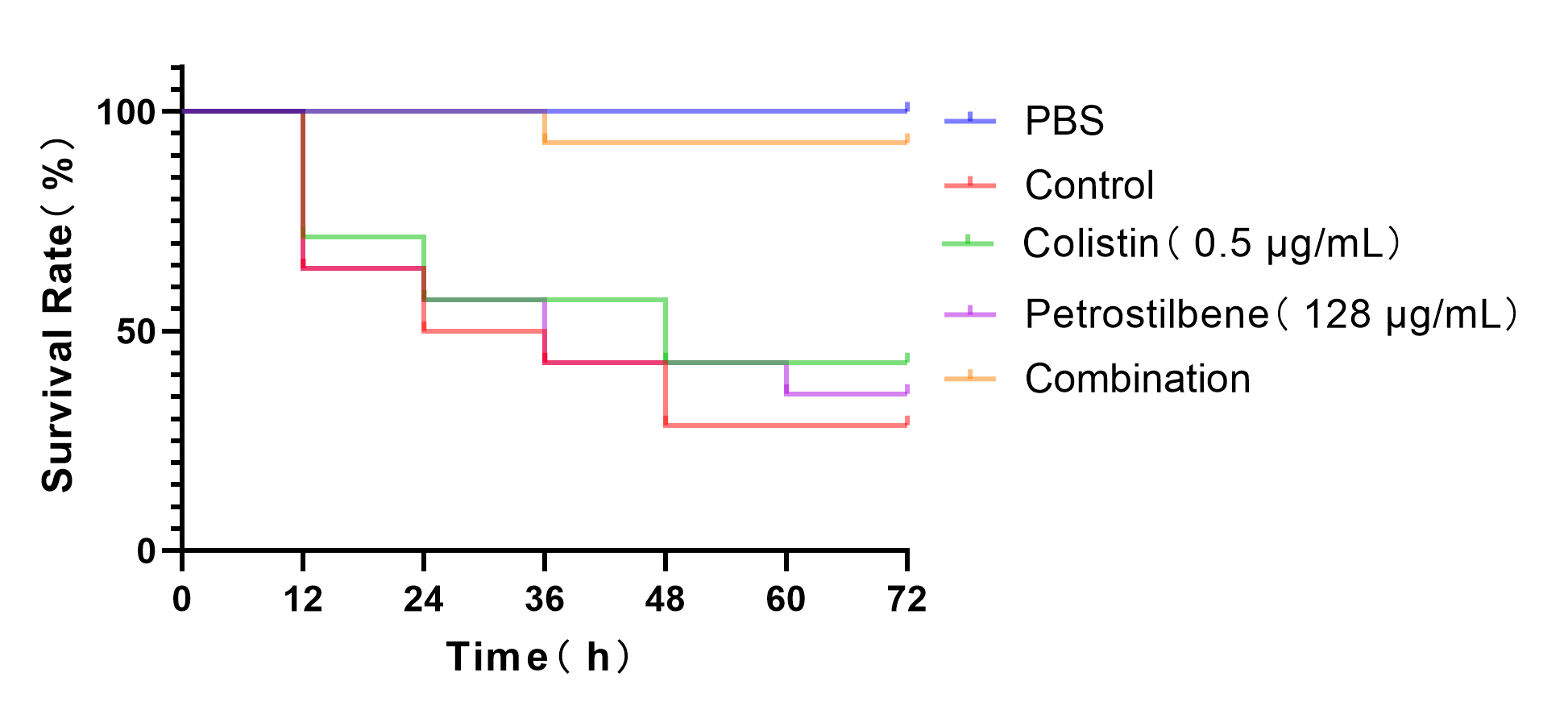


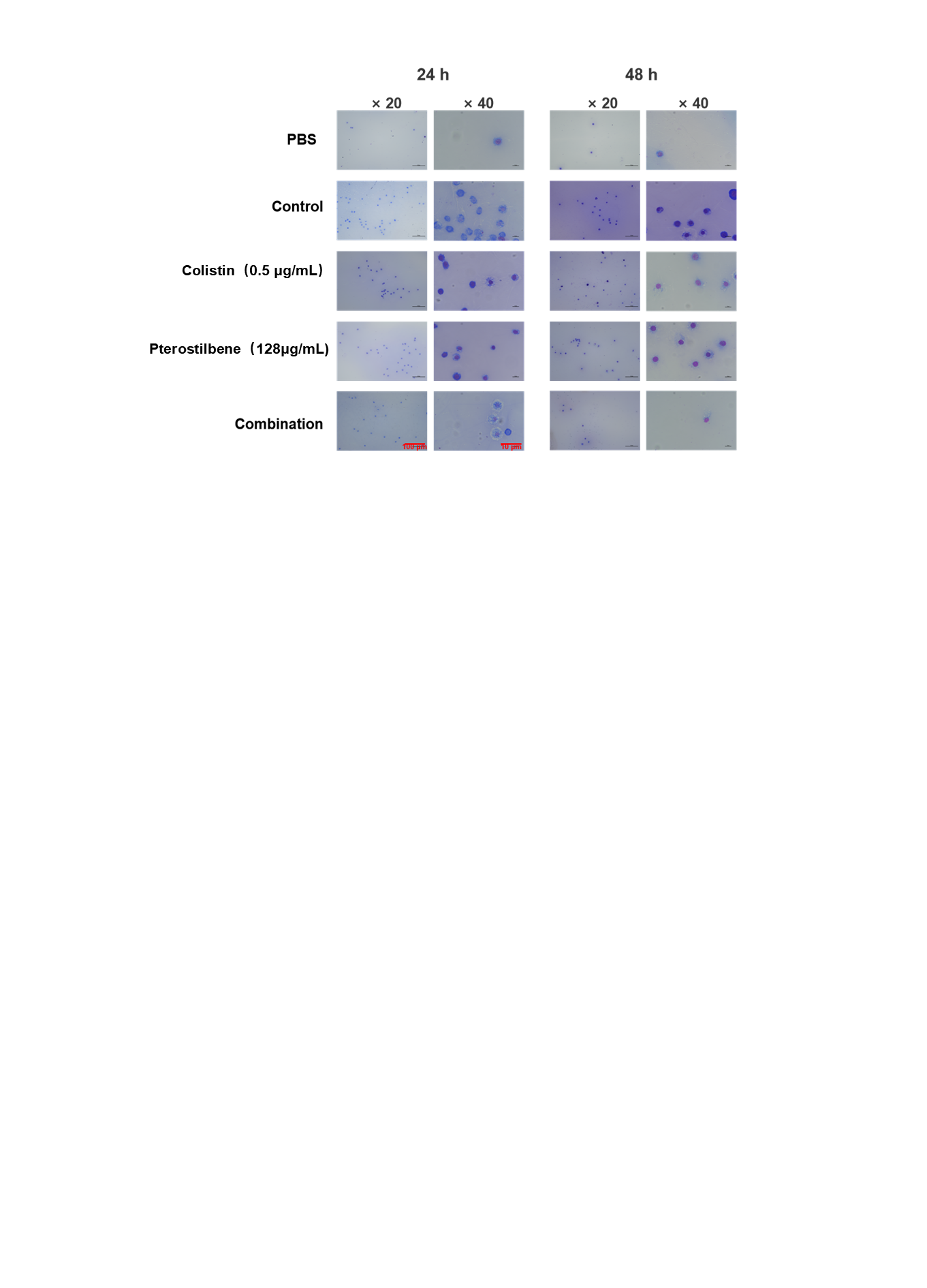
Figure S3. Survival curves of the *G. mellonella* infection model (n = 14, P < 0.001).

Figure S4. Giemsa staining of hemolymph smears from the *G. mellonella* infection model. Changes in the number of phagocytic cells in the hemolymph were observed under the microscope at 24 h and 48 h post-infection. (×20, scale bar = 100 μm; ×40, scale bar = 10 μm)


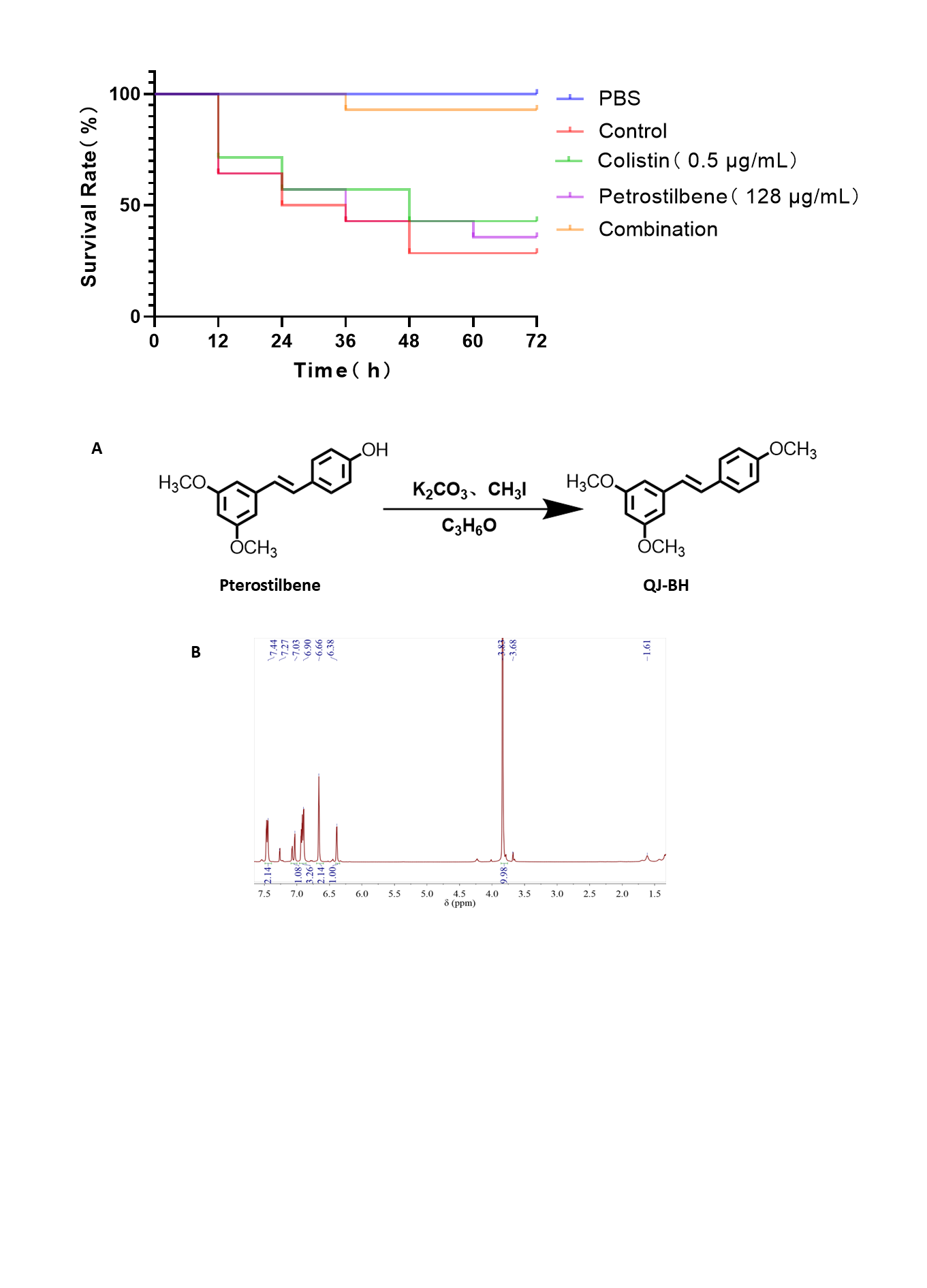


Figure S5. (A) Synthesis route to QJ-BH; (B) The ^1^H NMR spectrum of QJ-BH;


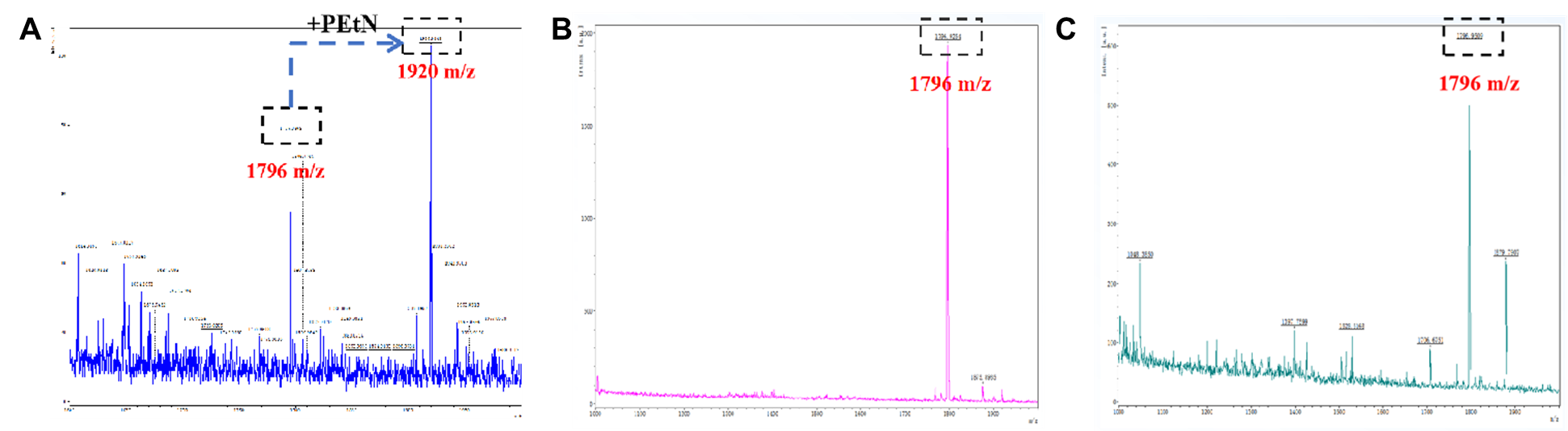


Figure S6. MALDI-TOF-MS analysis of lipid A from DH5α**⁺** strains.

(A) DH5α**⁺**-*mcr-3*; (B) DH5α*⁺*-*mcr-3*(E111A); (C) DH5α⁺-*mcr-3*(H380A)
